# Family carers experiences of compassion for self, other and receiving compassion

**DOI:** 10.1002/alz.088926

**Published:** 2025-01-09

**Authors:** Nuriye Kupeli, Claudia Cooper, Andrea Bruun, Laura Dillon, Pepsi Reilly, Nicola White, Elizabeth L Sampson

**Affiliations:** ^1^ University College London, London United Kingdom; ^2^ Queen Mary University of London, London United Kingdom; ^3^ East London NHS Foundation Trust, London United Kingdom; ^4^ University College London, London, London United Kingdom; ^5^ Alzheimer’s Society, London, London United Kingdom; ^6^ Royal London Hospital, East London NHS Foundation Trust, London United Kingdom

## Abstract

**Background:**

Family carers of people living with dementia provide care over many months or years with little to no formal support. Carers’ experiences of compassion for themselves and others including receiving compassion can influence their caregiving experience and the quality of care they provide. However, little is known about healthcare professionals (HCPs) understanding of how compassion is experienced by family carers and how best HCPs can support family carers to cultivate and maintain compassion for themselves and others.

**Methods:**

This study used qualitative interviews to explore family carers experience of compassion for themselves and others (including receiving compassion from others) while caring for someone living with dementia. Inductive thematic analysis was used to explore and report the barriers and facilitators to family carers experiences of compassion.

**Results:**

Interviews with ten family carers (nine current and one former carer) comprising of five daughters, three wives, one husband and one son were undertaken. Barriers and facilitators were identified for compassion for self, others and receiving compassion. While facilitators to compassion included having the opportunity to maintain an identify outside of caring role, positive memories, and shared understanding, experiences that made it difficult for carers to maintain compassion included the perception that one needs to sacrifice own needs and feelings of guilt and shame.

**Conclusions:**

Preliminary findings indicate that whilst carers of people living with dementia acknowledge that compassion is important, they indicate that the caring role could be a barrier to experiencing and maintaining compassion. Future research needs to focus on developing supportive interventions designed to addressing factors that may hinders carers abilities to cultivate and maintain compassion.

